# Development of an additive-controlled, SmI_2_-mediated stereoselective sequence: Telescoped spirocyclisation, lactone reduction and Peterson elimination

**DOI:** 10.3762/bjoc.9.163

**Published:** 2013-07-18

**Authors:** Brice Sautier, Karl D Collins, David J Procter

**Affiliations:** 1 University of Manchester, School of Chemistry, Oxford Road, Manchester M13 9PL, United Kingdom

**Keywords:** cyclisation, free radical, Peterson elimination, reduction, samarium, telescoped process

## Abstract

Studies on SmI_2_-mediated spirocyclisation and lactone reduction culminate in a telescoped sequence in which additives are used to “switch on” individual steps mediated by the electron transfer reagent. The sequence involves the use of two activated SmI_2_ reagent systems and a silicon stereocontrol element that exerts complete diastereocontrol over the cyclisation and is removed during the final stage of the sequence by Peterson elimination. The approach allows functionalised cyclopentanols containing two vicinal quaternary stereocentres to be conveniently prepared from simple starting materials.

## Introduction

Samarium diiodide (SmI_2_) has become an essential tool for chemists since its introduction by Kagan [1–2], efficiently mediating a wide range of reductive transformations [3]. The reagent’s versatility and the high degree of control usually observed in SmI_2_-mediated reactions make it the first choice for an array of reductive electron transfer processes [4–13]. Cyclisations are one of the most notable classes of transformation induced by SmI_2_ and have been widely employed in natural-product syntheses [8–10]. Importantly, fine tuning of the reagent’s reduction potential through the use of additives allows complex, polyfunctionalised starting materials to be manipulated selectively [3–16].

**Scheme 1:**
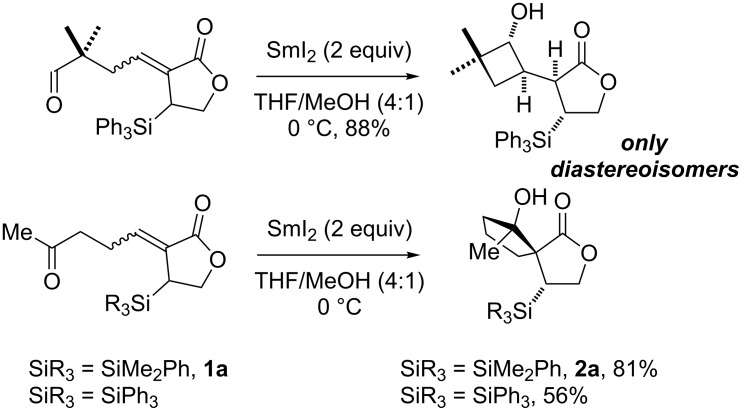
SmI_2_-mediated cyclisations directed by a C–Si bond.

Recently, we reported the use of a C–Si bond to control the stereochemical course of SmI_2_-mediated cyclisations. For example, complete diastereocontrol was achieved in the construction of cyclobutanols [13,17–22] and spirocyclopentanols (Scheme 1) [23–28]. The use of MeOH as an additive with SmI_2_ was key to the success of these cyclisations [24].

**Scheme 2:**
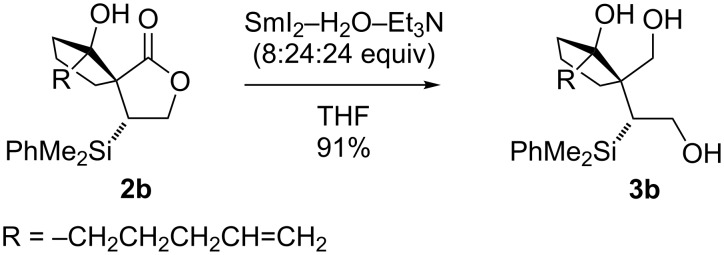
Reduction of a spirocyclic lactone using SmI_2_−H_2_O−Et_3_N.

In the case of spirocyclopentanol products **2**, further manipulation was hampered by their sensitivity to standard reductive conditions, and initially their reduction could only be achieved in two steps via the corresponding lactols [28]. An alternative solution for the manipulation of spirocyclopentanols **2** arose from our recent introduction of SmI_2_−H_2_O−amine [29–39] as a mild and efficient reagent system for the electron transfer reduction of carboxylic acid derivatives [40–42]. Pleasingly, SmI_2_−H_2_O−amine provided direct access to highly functionalised triols such as **3b** from spirocyclopentanol **2b** (Scheme 2) [42].

In this manuscript, we report studies on SmI_2_-mediated cyclisation and lactone reduction that culminate in a “telescoped” sequence, i.e., a sequence of steps carried out on a single reaction mixture by the sequential addition of various reagents. In the sequence, additives are used with SmI_2_ to “switch on” individual steps: spirocyclisation, lactone reduction and Peterson elimination allow rapid access to functionalised cyclopentanols, containing two vicinal quaternary stereocentres, from simple starting materials. The sequence involves the use of two activated SmI_2_ reagent systems, and a silicon stereocontrol element exerts complete diastereocontrol over the cyclisation and is removed during the final stage of the sequence.

## Results and Discussion

### Spirocyclisation

We first set out to examine the scope of the reductive-aldol spirocyclisation [23–27] directed by a C–Si bond, by varying the nature and functionalisation of the side chain. The ratio of **2b**/**4b** was optimized by adjusting the SmI_2_/MeOH ratio and the reaction time to minimise retro-aldol reaction and the formation of saturated ketolactone byproduct **4b** (Table 1). Lowering the amount of SmI_2_ and MeOH used and shortening the reaction time resulted in improved selectivity for spirolactone **2b**. We believe that spirolactones such as **2b** undergo retro-aldol fragmentation (to give products such as **4b**) upon prolonged exposure to Lewis acidic Sm(II)/(III)-species present in the reaction mixture.

**Table 1:** Optimisation of SmI_2_–mediated spirocyclisation conditions.

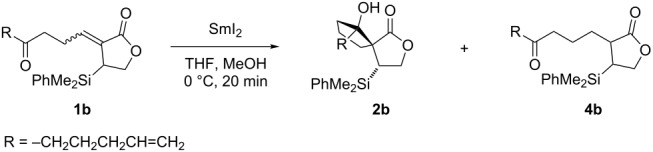				

Entry	SmI_2_^a^ (equiv)	MeOH (equiv)	SmI_2_/MeOH	**2b**/**4b**^b^

1	2.2	128	1:58	1.8:1
2	2.5	128	1:52	1.9:1
3	3	128	1:43	2.4:1
4	4	128	1:32	2.6:1
5	2.2	70	1:32	3.2:1
6	2.5	80	1:32	3.3:1
7	3	96	1:32	3.2:1
8^c^	2.5	80	1:32	4:1
9^c^	2.5	96	1:38	4:1

^a^0.1 M solution in THF. ^b^From ^1^H NMR of crude product mixture. ^c^Reaction time 3–5 min.

**Scheme 3:**
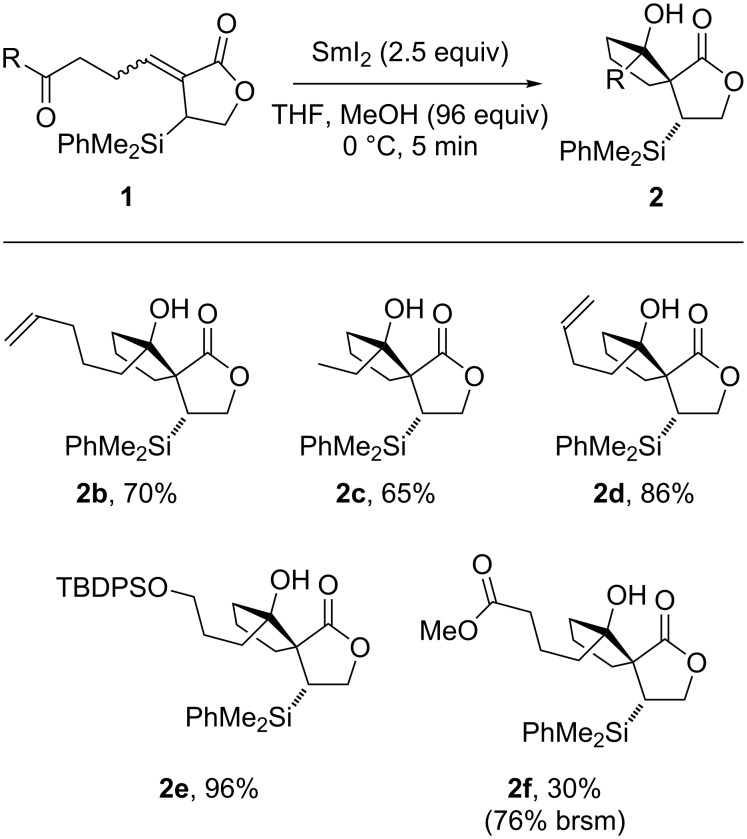
Stereoselective spirocyclisation of functionalised keto-lactone substrates directed by a C–Si bond.

Pleasingly, the process proved general, affording the desired spirocycles **2** in good yields and as single diastereoisomers with only small amounts of saturated ketolactone byproducts (cf. **4b**) observed. No byproducts arising from reaction of the additional functional groups present were formed (Scheme 3). Of particular note, keto-lactone **1f** bearing an ester-containing side chain gave the expected spirocycle **2f**, albeit with low conversion (unoptimized). As expected, no products arising from the reduction of the ester were observed [40–42].

### Telescoped spirocyclisation/lactone reduction

**Scheme 4:**
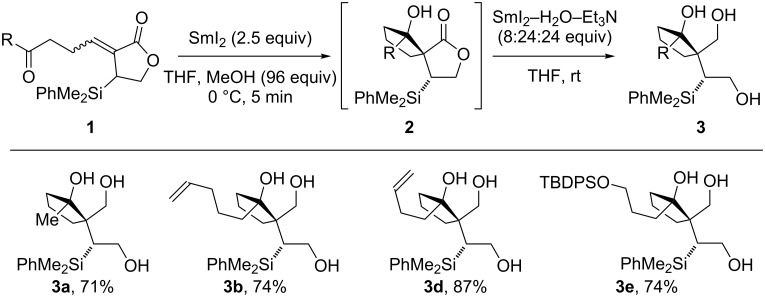
Telescoped stereoselective spirocyclisation/lactone reduction.

Although the reduction of the spirocycles **2** proceeds smoothly with SmI_2_−H_2_O−Et_3_N [40–42], we recognised the advantages of performing both SmI_2_-mediated steps in a telescoped fashion. The strongly coordinating H_2_O and amine additives used to activate SmI_2_ [29–42] in the second lactone reduction step suggested that this far more reducing system would tolerate the presence of samarium(III) salts and a less-activating additive (MeOH) from the first reduction step. Pleasingly, when subjected to the telescoped sequence, substrates **1** gave the desired triols **3** in comparable yields to those obtained from the stepwise process, without any need for further optimisation (Scheme 4).

The process is carried out by transferring the reaction mixture after the first reduction stage (SmI_2_–MeOH) to a preformed solution of SmI_2_−H_2_O−Et_3_N. The telescoped procedure proved robust and was scaled up to 1.2 g (3.5 mmol) without any drop in yield.

### Telescoped spirocyclisation/lactone reduction/Peterson elimination

With an efficient process combining spirocyclisation and lactone reduction in hand, we proposed that manipulation of the triol products by Peterson elimination [43–44] could be added to the telescoped sequence. Crucially, Peterson elimination of triols **3** would result in removal of the silicon stereocontrol element used to control the stereochemical course of C–C bond formation. In early studies, treatment of triol **3b** with *t*-BuOK gave vinyl cyclopentanol **5b** in moderate yield [45], but the reaction suffered from poor reproducibility. Following a screen of reaction conditions, moderate but consistent yields were obtained when eliminations were performed in an open vessel, using undried solvents.

**Scheme 5:**
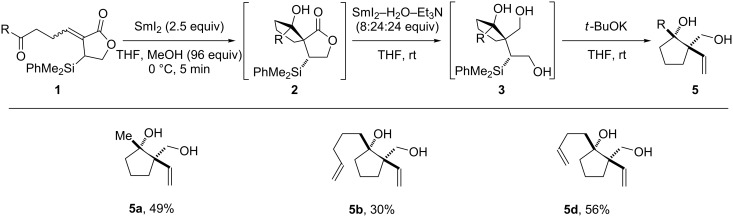
Telescoped stereoselective spirocyclisation/lactone reduction/Peterson elimination.

When combined with the spirocyclisation and lactone reduction sequence, the Peterson elimination gave diols **5**, with good overall yields comparable to those obtained for the stepwise process (Scheme 5).

We are currently exploring the use of the telescoped route to cyclopentanols **5** in an asymmetric approach [46] to the antitumor natural product pseudolaric acid B [47].

## Conclusion

In summary, we have developed a convenient, telescoped, three-step sequence to access functionalised cyclopentanols bearing two vicinal quaternary stereocentres from simple keto-lactone starting materials. The process involves the use of two activated SmI_2_ reagent systems and a silicon stereocontrol element that results in complete diastereocontrol and is removed in the final stage of the sequence. The procedure is scalable and the overall yields of the telescoped sequences compare well to the combined yields of the analogous stepwise processes. The use of additives to “switch on” individual steps in a particular sequence mediated by the same electron transfer reagent constitutes an exciting new opportunity for efficient synthesis.

## Supporting Information

File 1 General experimental procedures and characterisation data.
