# Supplementary material for: Development of an additive-controlled, SmI2-mediated stereoselective sequence: Telescoped spirocyclisation, lactone reduction and Peterson elimination
Source: Beilstein J Org Chem. 2013 Jul 18;9:1443–7. doi: 10.3762/bjoc.9.163 (PMC3740502; doi:10.3762/bjoc.9.163)
Supplement: File 1 — General experimental procedures and characterisation data. [file Beilstein_J_Org_Chem-09-1443-s001.pdf]

Supporting Information

for

Development of an additive-controlled, SmI_2-mediated stereoselective sequence: Telescoped spirocyclisation, lactone reduction and Peterson elimination

Brice Sautier, Karl D. Collins, David J. Procter*

Address: University of Manchester, School of Chemistry, Oxford Road, Manchester M13 9PL, United Kingdom

Email: David J. Procter - david.j.procter@manchester.ac.uk

* Corresponding author

General experimental procedures and characterisation data

General experimental 2

Preparation of samarium diiodide 2

Experimental 3

Literature procedures 3

General procedure A – Spirocyclisation 3

General procedure B – Telescoped spirocyclisation/lactone reduction 7

General procedure C – Telescoped spirocyclisation/lactone reduction/Peterson elimination 10

General experimental[1,2]

All experiments were performed under an atmosphere of nitrogen, using anhydrous solvents unless stated otherwise. Tetrahydrofuran was distilled from sodium/benzophenone and stored under nitrogen, and was deoxygenated by bubbling with nitrogen for ten minutes when used in conjunction with samarium diiodide. Triethylamine was distilled from calcium hydride and stored under nitrogen. Water when used in conjunction with samarium diiodide was distilled before being deoxygenated by bubbling with nitrogen for four hours. All other dry solvents were used as purchased from Sigma-Aldrich.

^{1}H NMR and ^{13}C NMR were recorded on 300, 400 and 500 MHz spectrometers, with chemical shift values being reported in ppm relative to residual chloroform (δ_H = 7.27 or δ_C = 77.2) as internal standards. All coupling constants (*J*) are reported in hertz (Hz).

Mass spectra were obtained using positive or negative electrospray (ES±) or electron ionization (EI±) methodology. Infrared spectra were recorded as evaporated films or neat using FTIR spectrometers. Melting points were measured on material obtained after column chromatography.

Column chromatography was carried out using 40–60 μ, 60 Å silica gel. Routine TLC analysis was carried out on aluminium sheets coated with silica gel 60 F254, 0.2 mm thickness. Plates were viewed using a 254 nm ultraviolet lamp and dipped in *para*-anisaldehyde or potassium permanganate.

Preparation of samarium diiodide

Samarium powder (1.2 g, 8.0 mmol, 1.2 equiv), diiodoethane (1.9 g, 6.7 mmol, 1.0 equiv), THF (55 mL) were added to a nitrogen-flushed, oven-dried round-bottom flask. The flask was covered with aluminium foil and the mixture was then stirred at room temperature for a minimum of 5 h (typically overnight). The solution was then left to settle for 1 h, titrated,[3] and used directly.

---

[1] Szostak, M.; Spain, M.; Procter, D. J. *J. Org. Chem.*, **2012**, *77*, 3049.
[2] For a detailed procedure on the preparation of SmI_2 and its use, see: Szostak, M.; Spain, M.; Procter, D. J. *Nature Protocols*, **2012**, *7*, 970.
[3] Dahlén, A.; Hilmersson, G. *Eur. J. Inorg. Chem.*, **2004**, 3020.

Experimental

Literature procedures

Substrates **1** were synthesised according to the procedure described by Harb *et al.*[4]

General procedure A – Spirocyclisation

To a stirred solution of SmI_2 (2.5 equiv, 0.1 M in THF) and MeOH (96 equiv) at 0 °C was added **1** (1.0 equiv in THF) and the reaction mixture was stirred for 5 min. The reaction was quenched by bubbling air through it. H_2O and tartaric acid (100 mg/mmol **1**) were then added, the aqueous phase was extracted with ethyl acetate (3×), and the combined organic phases were dried (Na_2SO_4 or $MgSO_4$) and concentrated in vacuo. Purification by column chromatography on silica gel eluting with 20% ethyl acetate in petroleum ether (40–60 °C) gave **2**.

***rac*-(4*R*,5*S*,6*S*)-4-(Dimethyl(phenyl)silyl)-6-hydroxy-6-methyl-2-oxaspiro[4.4]nonan-1-one (2a)**[4]

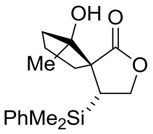

mp 71–74 °C;

ν_{max} (neat)/cm^{-1} 3523, 3436, 3276, 3068, 2360, 1747 (C=O), 1661, 1427, 1375, 1250, 1181, 1113, 1021, 833, 818;

^{1}H NMR (400 MHz, $CDCl_3$) δ ppm 0.39 (3 H, s, $SiCH_3$), 0.45 (3 H, s, $SiCH_3$), 1.26 (3 H, s, CH_3), 1.32–1.44 (1 H, 1 H from CH_2), 1.57–1.65 (1 H, m, 1 H from CH_2), 1.75 (1 H, ddd, *J* = 13.6, 10.3, 5.0 Hz, 1 H from CH_2), 1.87–2.02 (2 H, m, 2 × 1 H from CH_2), 2.05–2.15 (2 H, m, 1 H from $CH_{2,}$ SiCH), 4.16 (1 H, dd, *J* = 8.8, 6.1 Hz, 1 H from OCH_2), 4.39 (1 H, t, *J* = 8.8 Hz, 1 H from OCH_2), 7.33–7.45 (3 H, m, 3 × Ar-H), 7.45–7.55 (2 H, m, 2 × Ar-H);

^{13}C NMR (100 MHz, $CDCl_3$) δ ppm −4.2 ($SiCH_3$), −4.0 ($SiCH_3$), 19.1 (CH_2), 23.0 (CH_3), 28.7 (SiCH), 30.4 (CH_2), 37.7 (CH_2), 57.4 (C^q), 66.9 (OCH_2), 82.4 (C^qOH), 127.2 (2 × Ar-C), 128.7 (Ar-C), 132.6 (2 × Ar-C), 135.7 (Ar-C^q), 181.0 (C=O);

m/z (+ES) 327 ((M + Na), 100%); (Found (M + Na), 327.1402. $C_{17}H_{24}O_3$NaSi requires *M*, 327.1387).

---

[4] Harb, H. Y.; Collins, K. D.; Altur, J. V. G.; Bowker, S.; Campbell, L.; Procter, D. J. *Org. Lett.*, **2010**, *12*, 5446.

***rac*-(4*R*,5*S*,6*S*)-4-(Dimethyl(phenyl)silyl)-6-hydroxy-6-(pent-4-en-1-yl)-2-oxaspiro[4.4]nonan-1-one (2b)**

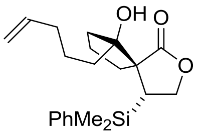

ν_{max} (neat)/cm^{-1} 3442 br. (OH), 3064, 2946, 2914, 2863, 1739 (C=O), 1427, 1372, 1261, 1191, 1111, 1029, 910, 832, 817, 736, 703;

^{1}H NMR (400 MHz, $CDCl_3$) δ 0.39 (3 H, s, $SiCH_3$), 0.47 (3 H, s, $SiCH_3$), 1.31–1.48 (5 H, m, C$H_2$$CH_2$CH=$CH_2$, 3 H from CH_2), 1.48–1.55 (1 H, m, 1 H from CH_2), 1.74–1.84 (2 H, m, CH_2), 1.85–1.97 (1 H, m, 1 H from CH_2), 2.00–2.08 (3 H, m, SiCH, CH_2CH=CH_2), 2.13 (1 H, ddd, *J* = 13.3, 10.8, 5.7 Hz, 1 H from CH_2), 3.74 (1 H, s, OH), 4.14 (1 H, dd, *J* = 9.0, 8.3 Hz, 1 H from OCH_2), 4.31 (1 H, dd, *J* = 8.3, 8.3 Hz, 1 H from OCH_2), 4.90–5.11 (2 H, m, CH=CH_2), 5.78 (1 H, ddt, *J* = 16.9, 10.2, 6.7 Hz, C*H*=CH_2), 7.32–7.46 (3 H, m, 3 × Ar-H), 7.47–7.52 (2 H, m, 2 × Ar-H);

^{13}C NMR (100 MHz, $CDCl_3$) δ −3.0 ($SiCH_3$), −2.7 ($SiCH_3$), 20.9 (CH_2), 23.3 (CH_2), 30.8 (SiCH), 31.8 (CH_2), 34.3 (*C*H_2CH=CH_2), 35.7 (CH_2), 36.4 (CH_2), 58.1 (C^q), 68.3 (OCH_2), 85.7 (C^qOH), 115.0 (CH=CH_2), 128.4 (2 × Ar-C), 130.0 (Ar-C), 133.7 (2 × Ar-C), 136.6 (Ar-C^q), 138.7 (C*H*=CH_2), 183.3 (C=O);

m/z (+ES) 381 ((M + Na), 100%); (Found (M + Na), 381.1850. $C_{21}H_{30}O_3NaSi$ requires *M*, 381.1857).

***rac*-(4*R*,5*R*,6*S*)-6-Ethyl-6-hydroxy-4-(dimethyl(phenyl)silyl)-2-oxa-spiro[4.4]nonan-1-one (2c)**

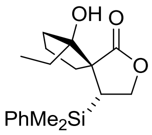

ν_{max} (neat)/cm^{-1} 3450, 2972, 2892, 1752 (C=O), 1709 (C=O), 1665, 1168, 1109, 1201, 994, 816;

^{1}H NMR (300 MHz, $CDCl_3$) δ ppm 0.41 (3 H, s, $SiCH_3$), 0.47 (3 H, s, $SiCH_3$), 0.94 (3 H, t, *J* = 7.3 Hz, CH_2CH_3), 1.30–1.40 (1 H, m, 1 H from CH_2), 1.40–1.48 (1 H, m, 1 H from CH_2), 1.52 (2 H, q, *J* = 7.3 Hz, C$H_2$$CH_3$), 1.71–1.87 (2 H, m, 2 × 1 H from CH_2), 1.87–2.00 (1 H, m, 1 H from CH_2), 2.06 (1 H, t, *J* = 8.0 Hz, SiCH), 2.10–2.23 (1 H, m, 1 H from CH_2), 3.61 (1 H, s, OH), 4.15 (1 H, dd, *J* = 9.0, 8.0 Hz, 1 H from OCH_2), 4.33 (1 H, t, *J* = 9.0 Hz, 1 H from OCH_2), 7.32–7.45 (3 H, m, 3 × Ar-H), 7.44–7.58 (2 H, m, 2 × Ar-H);

^{13}**C NMR** (75 MHz, $CDCl_3$) δ ppm −3.0 (2 × $SiCH_3$), 8.2 (CH_2*C*H_3), 20.6 (CH_2), 28.6 (*C*H_2CH_3), 30.4 (SiCH), 31.8 (CH_2), 35.7 (CH_2), 57.9 (C^q), 68.0 (CH_2O), 85.9 (C^qOH), 128.2 (2 × Ar-C), 129.8 (Ar-C), 133.6 (2 × Ar-C), 136.6 (Ar-C^q), 183.0 (C=O);
m/z (+ES) 341 ((M + Na), 100%), 357 (40) ; (Found (M + Na), 341.1547. $C_{18}H_{26}O_3NaSi$ requires *M*, 341.1543).

***rac*-(4*S*,5*S*,6*R*)-6-(But-3-en-1-yl)-4-(dimethyl(phenyl)silyl)-6-hydroxy-2-oxaspiro[4.4]nonan-1-one (2d)**

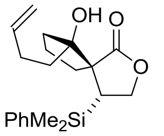

ν_{max} (neat)/cm^{-1} 3446 (br. OH), 3070, 2954, 2909, 1762 (C=O), 1738, 1428, 1373, 1253, 1171, 1111, 1028, 997, 912, 832, 815, 779, 735, 701, 652;
^{1}**H NMR** (400 MHz, $CDCl_3$) δ ppm 0.39 (3 H, s, $SiCH_3$), 0.47 (3 H, s, $SiCH_3$), 1.30–1.40 (1 H, m, 1 H from C*H_2*CH_2CH), 1.41–1.47 (1 H, m, 1 H from CH_2), 1.51–1.57 (2 H, m, CH_2), 1.75–1.85 (2 H, m, 1 H from CH_2, 1 H from C*H_2*CH=CH_2), 1.88–1.96 (1 H, m, 1 H from C*H_2*CH_2CH), 2.05–2.09 (2 H, m, 1 H from CH_2, CHSi), 2.11–2.18 (1 H, m, 1 H from C*H_2*CH=CH_2), 2.19–2.25 (1 H, m, 1 H from CH_2), 4.16 (1 H, dd, *J* = 9.1, 7.9 Hz, 1 H from CH_2O), 4.34 (1 H, dd, *J* = 9.1, 9.1 Hz, 1 H from CH_2O), 4.96 (1 H, dd, *J* = 10.2, 1.8 Hz, 1 H from CH=C*H_2*), 5.02 (1 H, dd, *J* = 17.0, 1.8 Hz, 1 H from CH=C*H_2*), 5.80 (1 H, ddt, *J* = 17.0, 10.2, 6.6 Hz, C*H*=CH_2), 7.38–7.42 (3 H, m, 3 × ArH), 7.48–7.53 (2 H, m, 2 × ArH);
^{13}**C NMR** (100 MHz, $CDCl_3$) δ ppm −3.3 ($SiCH_3$), −3.0 ($SiCH_3$), 20.6 (*C*H_2CH_2CH), 28.3 (CH_2), 30.4 (CHSi), 31.5 (*C*H_2CH=CH_2), 35.2 (CH_2), 35.8 (CH_2), 58.0 (C^q), 68.0 (CH_2O), 85.4 (C^qOH), 114.5 (CH=*C*H_2), 128.2 (2 × ArCH), 129.7 (ArCH), 133.5 (2 × ArCH), 136.4 (ArC^q), 138.6 (*C*H=CH_2), 182.8 (C=O);
m/z (ES+) 367 ((M + Na), 100%). (Found: (M + Na) 367.1714. $C_{20}H_{28}O_3SiNa$ requires *M*, 367.1700).

***rac*-(4*S*,5*S*,6*R*)-6-(3-((*tert*-Butyldiphenylsilyl)oxy)propyl)-4-(dimethyl(phenyl)silyl)-6-hydroxy-2-oxaspiro[4.4]nonan-1-one (2e)**

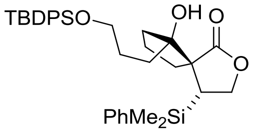

ν$_{max}$ (neat)/cm^{-1} 3438 (br. OH), 3070, 2953, 2929, 2857, 1763 (C=O), 1427, 1374, 1253, 1173, 1110, 1028, 998, 909, 821;

^{1}H NMR (400 MHz, $CDCl_3$) δ ppm 0.40 (3 H, s, $SiCH_3$), 0.46 (3 H, s, $SiCH_3$), 1.08 (9 H, s, 3 × CCH_3), 1.32–1.41 (1 H, m, 1 H from CH_2), 1.45–1.53 (1 H, m, 1 H from CH_2), 1.54–1.63 (3 H, m, C$H_2$$CH_2$OSi, 1 H from C$H_2$$CH_2$$CH_2$OSi), 1.65–1.74 (1 H, m, 1 H from C$H_2$$CH_2$$CH_2$OSi), 1.74–1.88 (2 H, m, 2 H from CH_2), 1.88–2.00 (1 H, m, 1 H from CH_2), 2.04 (1 H, t, *J* = 7.7 Hz, CHSi), 2.15 (1 H, ddd, *J* = 13.2, 10.7, 6.0 Hz, 1 H from CH_2), 3.68 (2 H, t, *J* = 5.7 Hz, CH_2OSi), 4.14 (1 H, dd, *J* = 9.1, 7.6 Hz, 1 H from CHCH_2O), 4.32 (1 H, t, *J* = 8.7 Hz, 1 H from CHCH_2O), 7.32–7.50 (11 H, m, 11 × ArH), 7.64–7.71 (4 H, m, 4 × ArH);

^{13}C NMR (100 MHz, $CDCl_3$) δ ppm −3.1 ($SiCH_3$), −3.0 ($SiCH_3$), 19.2 (C^q), 20.6 (CH_2), 26.9 (3 × CCH_3), 27.1 ($CH_2$$CH_2$$CH_2$OSi), 30.3 (CHSi), 31.7 (CH_2), 32.5 ($CH_2$$CH_2$OSi), 36.1 ($CH_2$), 58.0 ($C^q$), 64.3 (C$H_2$OSi), 68.0 (CH$CH_2$O), 85.4 ($C^q$OHs), 127.6 (4 × ArCH), 128.2 (2 × ArCH), 129.6 (2 × ArCH), 129.7 (ArCH), 133.5 (2 × ArCH), 133.8 (ArC^q), 133.8 (ArC^q), 135.5 (4 × ArCH), 136.6 (ArC^q), 182.9 (C=O).

rac-Methyl 4-((4*R*,5*S*,6*R*)-4-(dimethyl(phenyl)silyl)-6-hydroxy-1-oxo-2-oxaspiro[4.4]-nonan-6-yl)butanoate (2f)

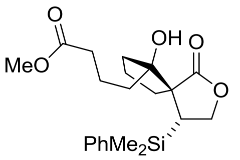

ν$_{max}$ (neat)/cm−1 2953, 1765 (C=O), 1737 (C=O), 1254, 1171, 1112, 1023, 998, 834, 819;

^{1}H NMR (400 MHz, $CDCl_3$) δ ppm 0.41 (3 H, s, $SiCH_3$), 0.48 (3 H, s, $SiCH_3$), 1.31 - 1.98 (9 H, m, 9H from CH_2), 2.13 (1 H, t, *J* = 8.4 Hz, SiCH), 2.27 - 2.40 (3 H, m, 3H from CH_2), 3.70 (3 H, s, OCH_3), 4.15 (1 H, t, *J* = 8.9 Hz, 1H from OCH_2), 4.27 - 4.37 (1 H, m, 1H from OCH_2), 7.34 - 7.60 (5 H, m, 5 × Ar-H);

^{13}C NMR (100 MHz, $CDCl_3$) δ ppm −3.1 ($SiCH_3$), −2.9 ($SiCH_3$), 19.4 (CH_2), 20.8 (CH_2), 30.5 (SiCH), 31.7 (CH_2), 34.1 (CH_2), 35.5 (CH_2), 36.5 (CH_2), 57.7 (C^q), 68.2 (OCH_2), 85.3 (C^qOH), 128.2 (2 × Ar-C), 129.8 (Ar-C), 133.6 (2 × Ar-C), 136.4 (Ar-Cq), 173.9 (CO_2CH_3), 183.3 (CO_2CH_2);

m/z (+ES) 413 ((M + Na), 35%) 523 (100); (Found (M + Na), 413.1761. $C_{21}H_{30}O_5$NaSi requires *M*, 413.1755).

General procedure B – Telescoped spirocyclisation/lactone reduction

To a stirred solution of SmI_2 (2.5 equiv, 0.1 M in THF) and MeOH (96 equiv) at 0 °C was added **1** (1.0 equiv in THF) and the reaction mixture was stirred for 5 min. The reaction mixture was quickly transferred by cannula into a preformed mixture of SmI_2 (8.0 equiv, 0.1 M in THF), Et_3N (24 equiv) and H_2O (24 equiv), and the resulting mixture was stirred at room temperature for 5 min. The reaction was quenched by opening the vessel to air. H_2O and tartaric acid (100 mg/mmol **1**) were then added, the aqueous phase was extracted with ethyl acetate (3×), and the combined organic phases were dried (Na_2SO_4 or $MgSO_4$) and concentrated in vacuo. Purification by column chromatography on silica gel eluting with 30% ethyl acetate in petroleum ether (40–60 °C) gave **3**.

***rac*-(1*S*,2*R*)-2-((*S*)-1-(Dimethyl(phenyl)silyl)-2-hydroxyethyl)-2-(hydroxymethyl)-1-methylcyclopentanol (3a)**

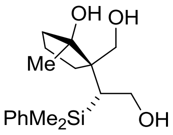

ν$_{max}$ (neat)/cm^{-1} 3284 (br. OH), 2957, 2876, 1427, 1372, 1304, 1248, 1145, 1110, 1054, 961, 912, 816, 777, 733, 699, 645;

^{1}H NMR (500 MHz, $CDCl_3$) δ ppm 0.46 (3 H, s, $SiCH_3$), 0.47 (3 H, s, $SiCH_3$), 1.20 (3 H, s, CCH_3), 1.17–1.23 (2 H, m, CH_2), 1.39–1.48 (1 H, m, 1 H from CH_2), 1.51–1.59 (1 H, m, 1 H from CH_2), 1.61 (1 H, br. d, *J* = 5.4 Hz, CHSi), 1.72 (1 H, ddd, *J* = 12.8, 9.9, 2.5 Hz, 1 H from CH_2), 2.02–2.11 (1 H, m, 1 H from CH_2), 3.63 (1 H, d, *J* = 12.5 Hz, 1 H from CCH_2OH), 3.85 (1 H, dd, *J* = 10.8, 5.5 Hz, 1 H from $CHCH_2OH$), 3.94 (1 H, dd, *J* = 12.5, 1.7 Hz, 1 H from CCH_2OH), 4.51 (1 H, dd, *J* = 10.8, 0.9 Hz, 1 H from $CHCH_2OH$), 7.33–7.38 (3 H, m, 3 × ArH), 7.56–7.61 (2 H, m, 2 × ArH);

^{13}C NMR (125 MHz, $CDCl_3$) δ ppm −0.2 ($SiCH_3$), 0.2 ($SiCH_3$), 17.8 (CH_2), 25.3 (CCH_3), 34.8 (CH_2), 37.0 (CHSi), 39.6 (CH_2), 50.6 (C^q), 61.4 ($CHCH_2OH$), 67.8 (CCH_2OH), 85.8 (C^qOH), 127.8 (2 × ArCH), 128.8 (ArCH), 133.9 (2 × ArCH), 140.1 (ArC^q).

rac-(1_S_,2_R_)-2-((_R_)-1-(Dimethyl(phenyl)silyl)-2-hydroxyethyl)-2-(hydroxymethyl)-1-(pent-4-en-1-yl)cyclopentanol (3b)[5]

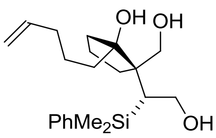

ν_{max} (neat)/cm^{-1} 3289 br. (OH), 2953, 2878, 1427, 1250, 1109, 1049, 910, 834, 818, 700;
^{1}H NMR (400 MHz, $CDCl_3$) δ 0.45 (3 H, s, $SiCH_3$), 0.47 (3 H, s, $SiCH_3$), 1.15 (2 H, dd, *J* = 8.9, 6.8 Hz, CH_2), 1.19–1.31 (1 H, m, 1 H from CH_2), 1.28–1.50 (2 H, m, 1 H from CH_2, 1 H from $CH_2CH_2CH{=}CH_2$), 1.48–1.63 (3 H, m, 1 H from $CH_2CH_2CH{=}CH_2$, CH_2), 1.65 (1 H, dd, *J* = 5.7, 1.6 Hz, SiCH), 1.74–1.88 (1 H, m, 1 H from CH_2), 1.98 (1 H, ddd, *J* = 13.4, 9.5, 2.3 Hz, 1 H from CH_2), 2.02–2.16 (2 H, m, $CH_2CH{=}CH_2$), 3.61 (1 H, d, *J* = 12.5 Hz, 1 H from C^qCH_2OH), 3.84 (1 H, dd, *J* = 10.8, 5.6 Hz, 1 H from $CHCH_2OH$), 3.96 (1 H, dd, *J* = 12.5, 1.6 Hz, 1 H from C^qCH_2OH), 4.46 (1 H, dd, *J* = 10.8, 1.3 Hz, 1 H from $CHCH_2OH$), 4.92–5.08 (2 H, m, $CH{=}CH_2$), 5.83 (1 H, ddt, *J* = 16.9, 10.2, 6.6 Hz, $CH{=}CH_2$), 7.31–7.38 (3 H, m, 3 × Ar-H), 7.55–7.62 (2 H, m, 2 × Ar-H);
^{13}C NMR (100 MHz, $CDCl_3$) δ 0.1 ($SiCH_3$), 0.4 ($SiCH_3$), 18.1 (CH_2), 22.7 (CH_2), 34.1 (CH_2), 34.5 (CH_2), 34.5 (CH_2), 34.8 (CH_2), 36.4 (SiCH), 51.5 (C^qCH_2OH), 61.7 ($CHCH_2OH$), 68.5 (C^qCH_2OH), 87.7 (C^qOH), 114.8 ($CH{=}CH_2$), 128.0 (2 × Ar-C), 129.0 (Ar-C), 134.0 (2 × Ar-C), 138.9 ($CH{=}CH_2$), 140.3 (Ar-C^q);
m/z (+ES) 301 (8%) 385 ((M + Na), 100); (Found (M + Na), 385.2169. $C_{21}H_{34}O_3NaSi$ requires *M*, 385.2170).

rac-(1_R_,2_R_)-1-(But-3-en-1-yl)-2-((_S_)-1-(dimethyl(phenyl)silyl)-2-hydroxyethyl)-2-(hydroxymethyl)cyclopentanol (3d)

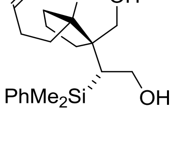

ν_{max} (neat)/cm^{-1} 3273 (br. OH), 3069, 2953, 2882, 1640, 1427, 1316, 1248, 1217, 1109, 1052, 1019, 957, 908, 816, 755, 732, 701, 646;
^{1}H NMR (400 MHz, $CDCl_3$) δ ppm 0.45 (3 H, s, $SiCH_3$), 0.48 (3 H, s, $SiCH_3$), 1.11–1.20 (2 H, m, CH_2), 1.24–1.43 (2 H, m, 1 H from CH_2CH_2CH, 1 H from CH_2), 1.48–1.62 (1 H, m, 1 H from CH_2),

[5] Szostak, M.; Collins, K. D.; Fazakerley, N. J.; Spain, M.; Procter, D. J. *Org. Biomol. Chem.* **2012**, *10*, 5820.

1.67 (1 H, d, *J* = 5.4 Hz, CHSi), 1.69–1.76 (1 H, m, 1 H from C*H*$_2$CH$_2$CH), 1.76–1.87 (1 H, m, 1 H from CH$_2$), 1.95–2.04 (1 H, m, 1 H from CH$_2$), 2.07–2.18 (1 H, m, 1 H from C*H*$_2$CH=CH$_2$), 2.19–2.30 (1 H, m, 1 H from C*H*$_2$CH=CH$_2$), 3.61 (1 H, d, *J* = 12.4 Hz, 1 H from CC*H*$_2$OH), 3.82 (1 H, dd, *J* = 10.5, 5.4 Hz, 1 H from CHC*H*$_2$OH), 3.95 (1 H, dd, *J* = 12.4, 1.3 Hz, 1 H from CC*H*$_2$OH), 4.43 (1 H, d, *J* = 10.5 Hz, 1 H from CHC*H*$_2$OH), 4.99 (1 H, ddt, *J* = 10.1, 1.7, 1.0 Hz, 1 H from CH=C*H*$_2$), 5.08 (1 H, dd, *J* = 17.0, 1.7 Hz, 1 H from CH=C*H*$_2$), 5.88 (1 H, ddt, *J* = 17.0, 10.1, 6.7 Hz, C*H*=CH$_2$), 7.32–7.39 (3 H, m, 3 × ArH), 7.56–7.62 (2 H, m, 2 × ArH);

^{13}**C NMR** (100 MHz, CDCl$_3$) δ ppm −0.2 (SiCH$_3$), 0.1 (SiCH$_3$), 17.8 (CH$_2$), 27.8 (*C*H$_2$CH=CH$_2$), 33.7 (CH$_2$), 34.2 (CH$_2$), 34.4 (*C*H$_2$CH$_2$CH), 36.0 (CHSi), 51.3 (C^q), 61.0 (CH*C*H$_2$OH), 68.0 (C*C*H$_2$OH), 87.3 (C^{q}OH), 114.6 (CH=*C*H$_2$), 127.7 (2 × ArCH), 128.7 (ArCH), 133.8 (2 × ArCH), 139.3 (*C*H=CH$_2$), 140.1 (ArC^q);

m/z (ES+) 371 ((M + Na), 100%), 372 (31). (Found: (M + Na) 371.2016. $C_{20}H_{32}O_3SiNa$ requires *M*, 371.2013).

***rac*-(1*R*,2*R*)-1-(3-((*tert*-Butyldiphenylsilyl)oxy)propyl)-2-((*S*)-1-(dimethyl(phenyl)silyl)-2-hydroxyethyl)-2-(hydroxymethyl)cyclopentanol (3e)**

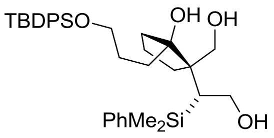

ν$_{max}$ (neat)/cm^{-1} 3290 (br. OH), 3069, 2956, 2928, 2856, 1472, 1427, 1250, 1110, 1047, 1008, 821, 735, 700, 614;

^{1}**H NMR** (400 MHz, CDCl$_3$) δ ppm 0.46 (3 H, s, SiCH$_3$), 0.50 (3 H, s, SiCH$_3$), 1.05 (9 H, s, 3 × CCH$_3$), 1.12–1.18 (1 H, m, 1 H from CH$_2$), 1.21–1.35 (3 H, m, CH$_2$, 1 H from CH$_2$), 1.42–1.92 (6 H, m, CHSi, 5 H from CH$_2$), 1.95–2.04 (1 H, m, 1 H from CH$_2$), 3.58 (1 H, d, *J* = 12.4 Hz, 1 H from CC*H*$_2$OH), 3.63–3.73 (2 H, m, CH$_2$C*H*$_2$OH), 3.81 (1 H, dd, *J* = 11.3, 5.6 Hz, 1 H from CHC*H*$_2$OH), 4.04 (1 H, dd, *J* = 12.4, 1.6 Hz, 1 H from CC*H*$_2$OH), 4.52 (1 H, dd, *J* = 11.3, 0.5 Hz, 1 H from CHC*H*$_2$OH), 7.32–7.46 (9 H, m, 9 × ArH), 7.58–7.62 (1 H, m, ArH), 7.63–7.70 (5 H, m, 5 × ArH);

^{13}**C NMR** (100 MHz, CDCl$_3$) δ ppm 0.0 (SiCH$_3$), 0.2 (SiCH$_3$), 18.1 (CH$_2$), 19.1 (C^q), 26.4 (CH$_2$), 26.8 (3 × C*C*H$_3$), 29.7 (CH$_2$), 32.8 (CH$_2$), 34.5 (CH$_2$), 37.0 (CHSi), 51.5 (C^q), 61.2 (CH*C*H$_2$OH), 64.9 (CH$_2$*C*H$_2$OH), 68.4 (C*C*H$_2$OH), 87.3 (C^{q}OH), 127.6 (2 × ArCH), 127.7 (4 × ArCH), 128.7 (ArCH), 129.8 (ArCH), 129.8 (ArCH), 133.1 (2 × ArCH), 133.1 (ArC^q), 133.8 (ArC^q), 133.9 (ArC^q), 135.6 (4 × ArCH).

General procedure C – Telescoped spirocyclisation/lactone reduction/Peterson elimination

To a stirred solution of SmI_2 (2.5 equiv, 0.1 M in THF) and MeOH (96 equiv) at 0 °C was added **1** (1.0 equiv in THF), and the reaction mixture was stirred for 5 min. The reaction mixture was quickly transferred by cannula into a preformed mixture of SmI_2 (8.0 equiv, 0.1 M in THF), Et_3N (24 equiv) and H_2O (24 equiv) at room temperature, and the resulting mixture was stirred for 5 min. The reaction was then quenched by opening the vessel to air. *t*-BuOK (40 equiv) was added portionwise over 20 min, and the reaction mixture was stirred for 20 min (completion monitored by TLC, eluting with 50% ethyl acetate in petroleum ether (40–60 °C)). H_2O and tartaric acid (100mg/mmol **1**) were added, the aqueous phase was extracted with ethyl acetate (3×), and the combined organic phases were dried (Na_2SO_4 or $MgSO_4$) and concentrated in vacuo. Purification by column chromatography on silica gel eluting with 30% ethyl acetate in petroleum ether (40–60 °C) gave **5**.

***rac*-(1*S*,2*R*)-2-(Hydroxymethyl)-1-methyl-2-vinylcyclopentanol (5a)**

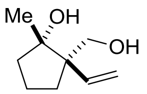

ν_{max} (neat)/cm^{-1} 3322 (br. OH), 2961, 2877, 1454, 1427, 1407, 1374, 1303, 1254, 1215, 1111, 1048, 1026, 961, 913, 833, 817, 736, 700, 657;

^{1}H NMR (400 MHz, $CDCl_3$) δ ppm 1.26 (3 H, s, CH_3), 1.62–1.87 (5 H, m, 5 H from CH_2), 1.98–2.08 (1 H, m, 1 H from CH_2), 3.71 (1 H, d, *J* = 11.3 Hz, 1 H from CH_2OH), 3.78 (1 H, d, *J* = 11.3 Hz, 1 H from CH_2OH), 5.10 (1 H, dd, *J* = 17.6, 1.3 Hz, 1 H from $CH{=}CH_2$), 5.14 (1 H, dd, *J* = 11.0, 1.3 Hz, 1 H from $CH{=}CH_2$), 5.86 (1 H, dd, *J*=17.6, 11.0 Hz, $CH{=}CH_2$);

^{13}C NMR (100 MHz, $CDCl_3$) δ ppm 19.1 (CH_2), 24.3 (CH_3), 29.2 (CH_2), 39.8 (CH_2), 54.3 (C^q), 66.1 (CH_2OH), 84.1 (C^qOH), 114.2 ($CH{=}CH_2$), 140.6 ($CH{=}CH_2$).

***rac*-(1*S*,2*R*)-2-(Hydroxymethyl)-1-(pent-4-en-1-yl)-2-vinylcyclopentanol (5b)**

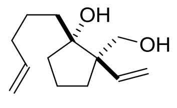

ν_{max} (neat)/cm^{-1} 3011 br. (OH), 3080, 2945, 2876, 1639, 1454, 1440, 1415, 1055, 1002, 908;

^{1}H NMR (400 MHz, $CDCl_3$) δ 1.24–1.39 (2 H, m, 1 H from $CH_2CH_2CH{=}CH_2$, 1 H from CH_2), 1.44–1.79 (7 H, m, 1 H from $CH_2CH_2CH{=}CH_2$, 3 × CH_2), 1.89–2.01 (2 H, m, $CH_2CH{=}CH_2$), 2.01–2.12 (1

H, m, 1 H from CH_2), 2.17–2.46 (1 H, br. s, OH), 2.58–2.90 (1 H, br. s, OH), 3.56 (1 H, d, *J* = 11.3 Hz, 1 H from $C\mathit{H}_2OH$), 3.76 (1 H, d, *J* = 11.3 Hz, 1 H from $C\mathit{H}_2OH$), 4.81–4.94 (2 H, $CH_2CH{=}C\mathit{H}_2$), 4.97 (1 H, dd, *J* = 12.7, 1.2 Hz, 1 H from $C^qCH{=}C\mathit{H}_2$), 5.01 (1 H, dd, *J* = 15.3, 1.2 Hz, 1 H from $C^qCH{=}C\mathit{H}_2$), 5.61–5.78 (2 H, m, 1 H from $C^qC\mathit{H}{=}CH_2$, $CH_2C\mathit{H}{=}CH_2$);
^{13}C NMR (100 MHz, $CDCl_3$) δ 19.5 (CH_2), 23.6 (CH_2), 29.7 (CH_2), 34.4 (CH_2), 36.1 (CH_2), 37.3 (CH_2), 55.3 ($\mathit{C}^qCH{=}CH_2$), 66.0 ($HOCH_2$), 86.6 (C^qOH), 114.2 ($CH{=}C\mathit{H}_2$), 114.8 ($CH{=}C\mathit{H}_2$), 138.8 ($C\mathit{H}{=}CH_2$), 140.8 ($C\mathit{H}{=}CH_2$).
m/z (+ES) 233 ((M + Na), 73%), 443 (2M + Na), 100); (Found (2M + Na), 233.1505. $C_{13}H_{22}O_2Na$ requires *M*, 233.1512).

***rac*-(1*R*,2*R*)-1-(But-3-en-1-yl)-2-(hydroxymethyl)-2-vinylcyclopentanol (5d)**

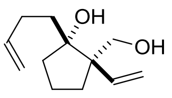

ν_{max} (neat)/cm^{-1} 3309 (br. OH), 3082, 2946, 2875, 1639, 1446, 1418, 1313, 1228, 1113, 1052, 1001, 911;
^{1}H NMR (500 MHz, $CDCl_3$) δ ppm 1.52 (1 H, ddd, *J* = 13.9, 11.0, 5.0 Hz, 1 H from $C\mathit{H}_2CH_2CH{=}CH_2$), 1.65–1.78 (5 H, m, 1 H from $C\mathit{H}_2CH_2CH{=}CH_2$, CH_2, 2 H from CH_2), 1.79–1.87 (1 H, m, 1 H from CH_2), 2.05–2.19 (2 H, m, 1 H from CH_2, 1 H from $C\mathit{H}_2CH{=}CH_2$), 2.23–2.33 (1 H, m, 1 H from $C\mathit{H}_2CH{=}CH_2$), 3.66 (1 H, d, *J* = 11.3 Hz, 1 H from $C\mathit{H}_2OH$), 3.86 (1 H, d, *J* = 11.3 Hz, 1 H from $C\mathit{H}_2OH$), 4.94–4.98 (1 H, m, 1 H from $CH_2CH{=}C\mathit{H}_2$), 5.05 (1 H, dd, *J* = 17.0, 1.7 Hz, 1 H from $CH_2CH{=}C\mathit{H}_2$), 5.05 (1 H, dd, *J* = 17.7, 1.1 Hz, 1 H from $CCH{=}C\mathit{H}_2$), 5.10 (1 H, dd, *J* = 11.0, 1.1 Hz, 1 H from $CCH{=}C\mathit{H}_2$), 5.79 (1 H, dd, *J* = 17.7, 11.0 Hz, $CC\mathit{H}{=}CH_2$), 5.86 (1 H, ddt, *J* = 17.0, 10.2, 6.6 Hz, $CH_2C\mathit{H}{=}CH_2$);
^{13}C NMR (125 MHz, $CDCl_3$) δ ppm 19.3 (CH_2), 28.7 ($\mathit{C}H_2CH{=}CH_2$), 29.5 (CH_2), 35.6 ($\mathit{C}H_2CH_2CH{=}CH_2$), 36.8 ($CH_2$), 55.0 ($C^q$), 65.7 ($CH_2OH$), 86.4 ($C^qOHs$), 114.0 ($CH_2CH{=}\mathit{C}H_2$), 114.5 ($CCH{=}\mathit{C}H_2$), 139.3 ($C\mathit{C}H{=}CH_2$), 140.5 ($CH_2\mathit{C}H{=}CH_2$);
m/z (ES+) 119 (50%), 161 (85), 219 ((M + Na), 100). (Found: (M + Na) 219.1364. $C_{12}H_{20}O_2Na$ requires *M*, 219.1356).
